# Coenzyme Q10 Attenuates High Glucose-Induced Endothelial Progenitor Cell Dysfunction through AMP-Activated Protein Kinase Pathways

**DOI:** 10.1155/2016/6384759

**Published:** 2015-11-22

**Authors:** Hsiao-Ya Tsai, Chih-Pei Lin, Po-Hsun Huang, Szu-Yuan Li, Jia-Shiong Chen, Feng-Yen Lin, Jaw-Wen Chen, Shing-Jong Lin

**Affiliations:** ^1^Institute of Clinical Medicine, National Yang-Ming University, Taipei, Taiwan; ^2^Department of Pathology and Laboratory Medicine, Taipei Veterans General Hospital, Taipei, Taiwan; ^3^Department of Biotechnology and Laboratory Science in Medicine and Institute of Biotechnology in Medicine, National Yang-Ming University, Taipei, Taiwan; ^4^Division of Cardiology, Taipei Veterans General Hospital, Taipei, Taiwan; ^5^Cardiovascular Research Center, National Yang-Ming University, Taipei, Taiwan; ^6^Division of Nephrology, Department of Medicine, Taipei Veterans General Hospital, Taipei, Taiwan; ^7^Institute and Department of Pharmacology, National Yang-Ming University, Taipei, Taiwan; ^8^Department of Internal Medicine, School of Medicine, College of Medicine, Taipei Medical University, Taipei, Taiwan; ^9^Department of Medical Research, Taipei Veterans General Hospital, Taipei, Taiwan

## Abstract

Coenzyme Q10 (CoQ10), an antiapoptosis enzyme, is stored in the mitochondria of cells. We investigated whether CoQ10 can attenuate high glucose-induced endothelial progenitor cell (EPC) apoptosis and clarified its mechanism. EPCs were incubated with normal glucose (5 mM) or high glucose (25 mM) enviroment for 3 days, followed by treatment with CoQ10 (10 *μ*M) for 24 hr. Cell proliferation, nitric oxide (NO) production, and JC-1 assay were examined. The specific signal pathways of AMP-activated protein kinase (AMPK), eNOS/Akt, and heme oxygenase-1 (HO-1) were also assessed. High glucose reduced EPC functional activities, including proliferation and migration. Additionally, Akt/eNOS activity and NO production were downregulated in high glucose-stimulated EPCs. Administration of CoQ10 ameliorated high glucose-induced EPC apoptosis, including downregulation of caspase 3, upregulation of Bcl-2, and increase in mitochondrial membrane potential. Furthermore, treatment with CoQ10 reduced reactive oxygen species, enhanced eNOS/Akt activity, and increased HO-1 expression in high glucose-treated EPCs. These effects were negated by administration of AMPK inhibitor. Transplantation of CoQ10-treated EPCs under high glucose conditions into ischemic hindlimbs improved blood flow recovery. CoQ10 reduced high glucose-induced EPC apoptosis and dysfunction through upregulation of eNOS, HO-1 through the AMPK pathway. Our findings provide a potential treatment strategy targeting dysfunctional EPC in diabetic patients.

## 1. Introduction

Diabetes is a metabolic disease clinically expressed by chronic hyperglycemia that has been extensively reported to be linked to several micro- and macrovascular diseases that significantly impair the quality of life. Among diabetic vascular complications, foot ulcers represent the first cause of hospitalization in diabetic patients and a significant cause of health care costs [1]. Despite promising therapeutic strategies, morbidity and mortality in diabetic patients with peripheral artery disease have not been markedly improved over the last decade. Clinical studies have indicated that diabetic patients have impaired endothelial function and are prone to suffer from severe organ damage due to poor collateral vessel formation in response to tissue ischemia [2]. Accumulating evidence suggests that circulating endothelial progenitor cells (EPCs) are mainly derived from the monocyte/macrophage lineage, and are capable of forming new blood vessels in ischemic tissues through a process of vasculogenesis [3, 4]. It has been reported that in patients with diabetes the numbers and function of circulating EPCs are decreased, although the mechanisms underlying this decrease are poorly understood [5, 6]. Clinical and experimental studies have indicated that micro- or macrovascular complications associated with diabetes may be due to the reduced count and impaired functionality of circulating EPCs [5, 6].

Coenzyme Q (CoQ10), also known as ubiquinone, acts as an electron transport carrier from complex I or complex II to complex III within the inner mitochondrial membrane and exerts an important role in maintaining bioenergy homeostasis in mitochondria [7, 8]. Additionally, CoQ10 is also an antioxidant which neutralizes free radicals and inhibits cellular apoptosis [7]. There is a considerable amount of data suggesting that supplementation of CoQ10 has beneficial effects on cardiovascular disease, metabolic syndrome, and diabetes by improvement of mitochondrial membrane potential and counteracts oxidative stress in myocytes [9, 10]. Recent reports have also shown that CoQ10 augments endothelial function of type 2 diabetic patients and ischemic heart disease [11, 12]. However, whether treatment with CoQ10 improves dysfunctional EPC and prevents apoptosis in high glucose conditions remains unclear. In this study, we aim to investigate whether administration of CoQ10 could attenuate high glucose-induced apoptosis of EPCs and whether nitric oxide (NO) could be involved in the corresponding signaling pathway in this process. Our findings provide novel evidence that CoQ10 could be a potential therapeutic strategy to diminish ischemia-induced tissue damage by enhancement of EPC function in diabetic patients.

## 2. Materials and Methods

### 2.1. Cell Culture and Reagents

Total mononuclear cells (MNCs) were isolated by density gradient centrifugation with Histopaque-1077 (1.077 g/mL, Sigma) from peripheral blood of healthy young volunteers. Briefly, 5 × 10^6^ MNCs were seeded in EGM-2MV medium (Cambrex, East Rutherford, NJ, USA), with supplements (hydrocortisone, human epidermal growth factor, R^3^-insulin-like growth factor 1, human fibroblast growth factor, vascular endothelial growth factor (VEGF), gentamicin, amphotericin B, vitamin C, and 20% fetal bovine serum) on 0.1% fibronectin-coated plate. The medium was changed after every four days of culture, and EPCs appeared within 7–15 days after the start of the MNC culture. EPCs were used passage numbers 3 through 7. Additionally, we identified the EPCs by the antibodies CD34, CD133, KDR, CD31 (Santa Cruz), and vWF (Neomarkers) using immunofluorescence. The fluorescent images were obtained by a laser scanning confocal microscope. Coenzyme Q10 was purchased from Sigma, dissolved in 0.04% Lutrol F127, and then diluted in culture medium.

### 2.2. EPC Viability

After cells were cultured with different glucose conditions for 4 days or CoQ10 for 1 day, EPCs were treated with MTT (0.5 mg/mL, Sigma). The cells were lysed with dimethyl sulfoxide and measured at 550/650 nm.

### 2.3. EPC Migration

The migratory function of the EPCs was evaluated by a modified Boyden chamber (Transwell, Costar). Briefly, 4 × 10^4^ EPCs were placed in the upper chambers of transwell plates with serum-free endothelial growth medium. In the lower chambers, stromal cell-derived factor 1 (SDF-1) (50 ng/mL) was supplemented to the medium placed at 37°C incubation. After incubation for 24 hours, the membrane of chamber was washed by PBS twice and stained using lectin-FITC (UEA-1 lectin, Sigma). Then the upper membrane side was scraped with a cotton ball and fixed with 2% paraformaldehyde. The migrated cells in lower membrane side were counted by 6 random high-power (×100) microscopic fields by fluorescence microscopy.

### 2.4. EPC Senescence

EPCs aging was evaluated with a Senescence Cell Staining kit (Sigma, USA). The EPCs were fixed with 2% formaldehyde and 0.2% glutaraldehyde for 6 min. Then the cells were incubated at 37°C without CO_2_ in fresh X-gal staining solution (1 mg/mL X-gal, 5 mM potassium ferricyanide, and 2 mM MgCl_2_; pH6). After 6–8 hours, *β*-galactosidase-positive cells appeared (green). The level of senescence of EPCs was evaluated by calculating relative percentages of green-stained and total cells.

### 2.5. Mitochondrial Apoptosis

Mitochondrial apoptosis was determined by JC-1 assay (BD Pharmingen). The EPCs were harvested by trypsinization and incubated with JC-1. After 15 min, the cells were washed with PBS twice. Apoptosis was detected by a change in JC-1-labeled fluorescence from red to green with flow cytometer and analyzed with Cell Quest Alias software, as described in the literature [13].

### 2.6. Mitochondrial Function

Mitochondrial function was determined by measurement of mitochondrial membrane potential via rhodamine 123 (Rh123), as described in [14]. After treatment with high glucose or CoQ10, the cells were stained with Rh123 (5 mM, Sigma) and incubated at 37°C for 30 min. The intensity of fluorescence (relative fluorescence units) was measured at excitation wavelength 485-nm and emission wavelength 530-nm by a fluorescence microplate reader.

### 2.7. Nitric Oxide (NO) and Reactive Oxygen Species (ROS) Production

NO production was determined by staining with 3-amino,4-aminomethyl-2′,7′-difluorofluorescein (DAF-FM) diacetate (10 *μ*M, Molecular Probes) for 30 min. The intensity of fluorescence (relative fluorescence units) was evaluated at excitation wavelength 495-nm and emission wavelength 515-nm by a fluorescence microplate reader.

ROS was determined by H_2_O_2_ detection using 2′,7′-dichlorodihydrofluorescein diacetate (DCFH-DA 20 *μ*M, Molecular Probes) as a probe. The intensity of fluorescence was evaluated at excitation wavelength 485-nm and emission wavelength 530-nm by a fluorescence microplate reader.

### 2.8. Western Blotting

EPCs were lysed in protein lysis buffer (62.5 mM Tris-HCl, 2% SDS, 10% glycerol, 1 mM PMSF, and 1 *μ*g/mL aprotinin, pepstatin, and leupeptin). The proteins were separated by SDS-PAGE and transferred to PVDF membrane. Membranes were probed with antibodies against *β*-actin, eNOS, phosphorylated eNOS (Millipore, Billerica, MA, USA), Akt, phosphorylated Akt (Cell Signaling, Denvers, MA), AMPK, phosphorylated AMPK (Sigma, St. Louis, MO, USA), activated-caspase 3, Bcl-2, and HO-1 (Cell Signaling, Danvers, MA). The protein blots were detected by chemiluminescence detection using ImageQuant LAS 400.

### 2.9. Animals

Nude mice were purchased from the National Laboratory Animal Center, Taiwan. This animal study conforms to the Guide for the Care and Use of Laboratory Animals published by the US National Institutes of Health (NIH Publication 1996) and all experimental procedures involving the animals received the approval of the Institutional Animal Care Committee of National Yang-Ming University (Taipei, Taiwan). Experimental mice received unilateral hindlimb surgery to induce ischemia, which involved excision of the right femoral artery as previously described [15]. The femoral artery was ligated at the proximal and distal portions. The blood perfusion of hindlimb was measured by a laser Doppler perfusion imaging system (Moor Instruments Limited, Devon, UK). At 24 hours after surgery, EPCs, high glucose-cultured EPCs, and high glucose combined with CoQ10-cultured EPCs were labeled with PKH26 (2 nM, Sigma) for 5 min. The PKH26-labeled cells (1 × 10^5^) were injected into the ischemia limbs of operated nude mice. The control animals received the same volume of normal medium without EPCs.

### 2.10. Statistical Analysis

Statistical analyses were executed using SPSS software (version 14; SPSS, Chicago, IL, USA). Unpaired Student's *t*-test or analysis of variance was used to evaluate comparisons between groups. Significance was attained when a *P* value was less than 0.05.

## 3. Results

### 3.1. Characterization of Human EPC

**Figure 1:**
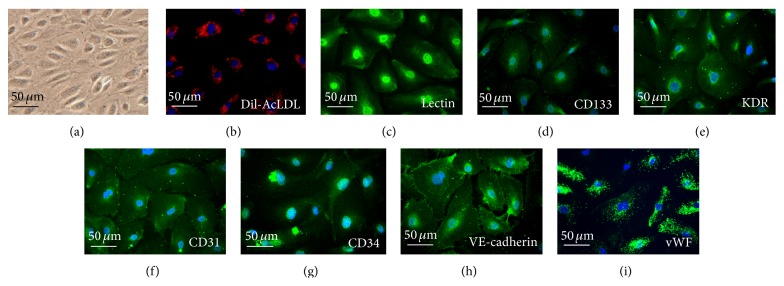
Morphology and characterization of EPCs from peripheral blood. (a) Peripheral blood mononuclear cells (MNCs) were plated on a fibronectin-coated culture dish on the fourteenth day. EPCs were also characterized by immunofluorescence staining for the expression of (b) DiI-AcLDL, (c) lectin, (d) CD133, (e) kinase insert domain receptor (KDR), (f) platelet/endothelial cell adhesion molecule-1 (CD31), (g) CD34, (h) VE-cadherin, and (i) Von Willebrand factor (vWF). Cells were counterstained with 4′,6-diamidino-2-phenylindole (DAPI) for the nuclei (blue).

As shown in Figure 1, MNCs were cultured on a fibronectin-coated dish on the fourteenth day (a). Most cells expressed DiI-acLDL uptake simultaneously (b) and fluorescein isothiocyanate UEA-1 (lectin, green (c)) binding. Then EPCs were characterized by immunofluorescence detection of CD133 (d), KDR (e), CD31 (f), CD34 (g), VE-cadherin (h), and vWF (i).

### 3.2. Effects of CoQ10 on EPC Viability, Migration, and Senescence in High Glucose Conditions In Vitro

**Figure 2:**
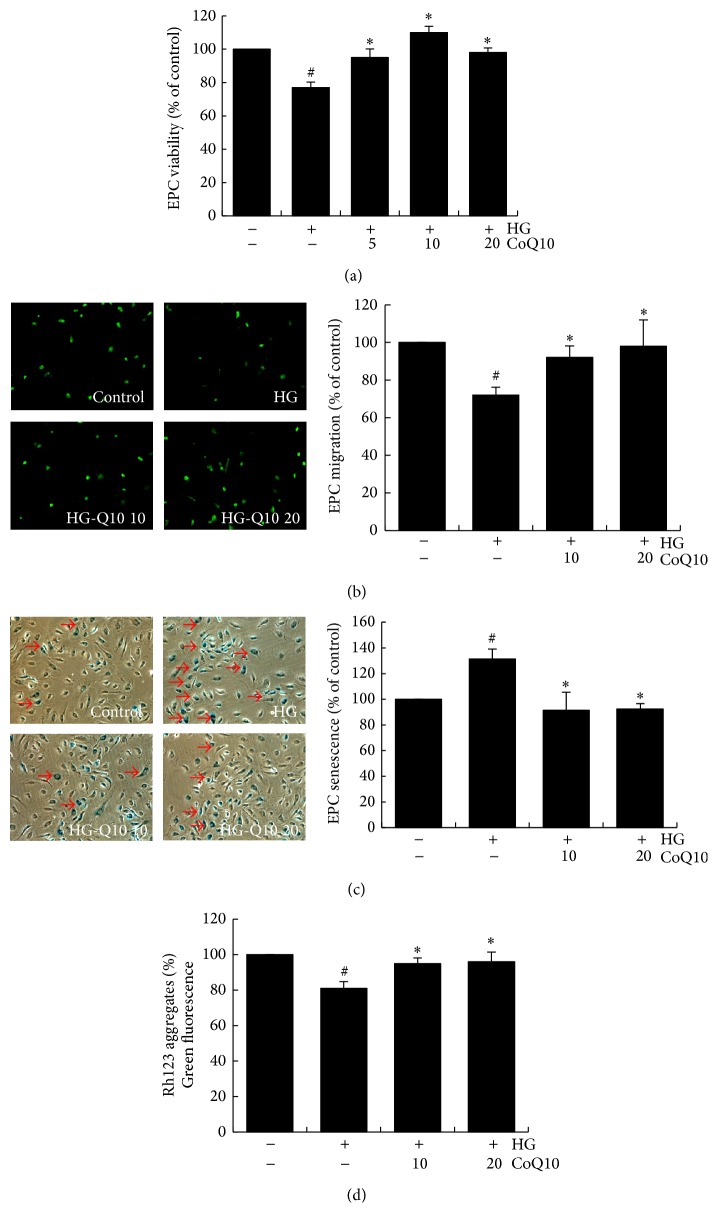
Effects of CoQ10 on EPC viability, migration, senescence, and mitochondrial function under high glucose conditions. Cells were cultured with glucose (25 mM) for 3 days, followed by treatment with CoQ10 (5 *μ*M, 10 *μ*M, and 20 *μ*M) for 24 hr. (a) EPC viability was analyzed by MTT assay. (b) A Boyden chamber assay was used with SDF-1 as chemoattractive factor for EPC migration. The migrated cells were stained with fluorescein isothiocyanate UEA-1 (lectin) (green) and counted under the fluorescence microscope. (c) EPC senescence was analyzed by senescence-associated acidic-*β*-galactosidase activity assay. (d) Cell mitochondrial function was measured by staining with rhodamine 123- (Rh123-) derived green fluorescence 5 mM for 20 min. Data are mean ± SE; *n* = 6; ^#^ *P* < 0.05 versus control (5 mM glucose); ^*∗*^ *P* < 0.05 versus high glucose (HG).

To clarify the effects of CoQ10 on high glucose-induced viability and migratory function, MTT and modified Boyden chamber assays were used to evaluate cell viability and migration. EPCs were cultured for 4 days in high glucose medium (25 mM) with and without the indicated concentrations of CoQ10 (5–20 *μ*M). High glucose environment decreased cell viability and attenuated EPC migration by 25% and 30%, respectively. However, treatment with CoQ10 significantly improved EPC viability and migration in high glucose conditions (Figures 2(a) and 2(b)).

We further examined whether CoQ10 improves EPC senescence under high glucose condition. Compared with the control group, EPCs incubated with high glucose showed a significant increase in senescence (*β*-galactosidase-positive cells) by 28%. As shown in Figure 2(c), administration of CoQ10 for 24 hours attenuated high glucose-induced senescence by 30%.

### 3.3. CoQ10 Recovered High Glucose-Suppressed Mitochondrial Function of EPCs

Mitochondrial function was analyzed by measurement of mitochondrial membrane potential. Rhodamine 123 (Rh123) emits green fluorescence and localizes in mitochondria and was used to determine mitochondrial membrane potential by accumulation of green fluorescence. As shown in Figure 2(d), high glucose reduced mitochondrial membrane potential of EPCs by 20%; however, treatment with CoQ10 significantly reversed this mitochondrial effect.

### 3.4. CoQ10 Improved High Glucose-Induced EPCs Apoptosis

**Figure 3:**
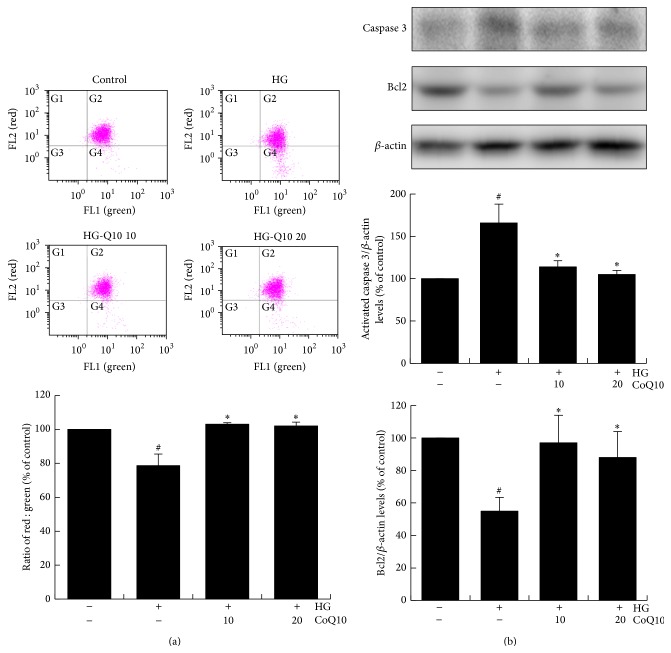
Effect of CoQ10 on EPC apoptosis under high glucose conditions. EPCs were incubated with CoQ10 (10 *μ*M) for 24 hrs in high glucose medium. (a) Mitochondrial apoptosis was detected by JC-1 assay by flow cytometry. Loss of mitochondrial membrane potential (ΔΨ_m_) was performed by the change in JC-1-derived fluorescence from red to green. The ratio of red/green fluorescence represented ΔΨ_m_ in EPCs. (b) Expression of activated-caspase 3 and Bcl-2 protein levels were assessed by western blot analysis. Data are mean ± SE; *n* = 4; ^#^ *P* < 0.05 versus control (5 mM glucose); ^*∗*^ *P* < 0.05 versus high glucose (HG).

During cellular apoptosis, disruption of the mitochondrial membrane potential (ΔΨ_m_) is one of the earliest intracellular events. To elucidate mitochondrial apoptosis of EPCs, JC-1 assay was used to analyze cellular apoptosis by the change in JC-1-derived fluorescence from red to green. Therefore, decline of mitochondrial membrane potential during apoptosis was represented by a decrease in red fluorescence intensity. The data shows that mitochondrial membrane potential (ΔΨ_m_) of high glucose-cultured EPCs fell by 22% and administration of CoQ10 reversed the mitochondrial membrane potential by 25%. Therefore, CoQ10 recovered mitochondrial apoptosis in high glucose-stimulated EPCs (Figure 3(a)).

To further assess the effect of CoQ10 on antiapoptosis capacity in EPCs, we determined the expression of associated apoptosis proteins. The results showed that high glucose increased activated-caspase 3 and decreased Bcl-2 expressions; however, treatment with CoQ10 (10 *μ*M and 20 *μ*M) significantly inhibited activated-caspase 3 and increased Bcl-2 expressions in high glucose-cultured EPCs (Figure 3(b)).

### 3.5. CoQ10 Activates AMPK Pathway of EPCs in High Glucose Conditions In Vitro

**Figure 4:**
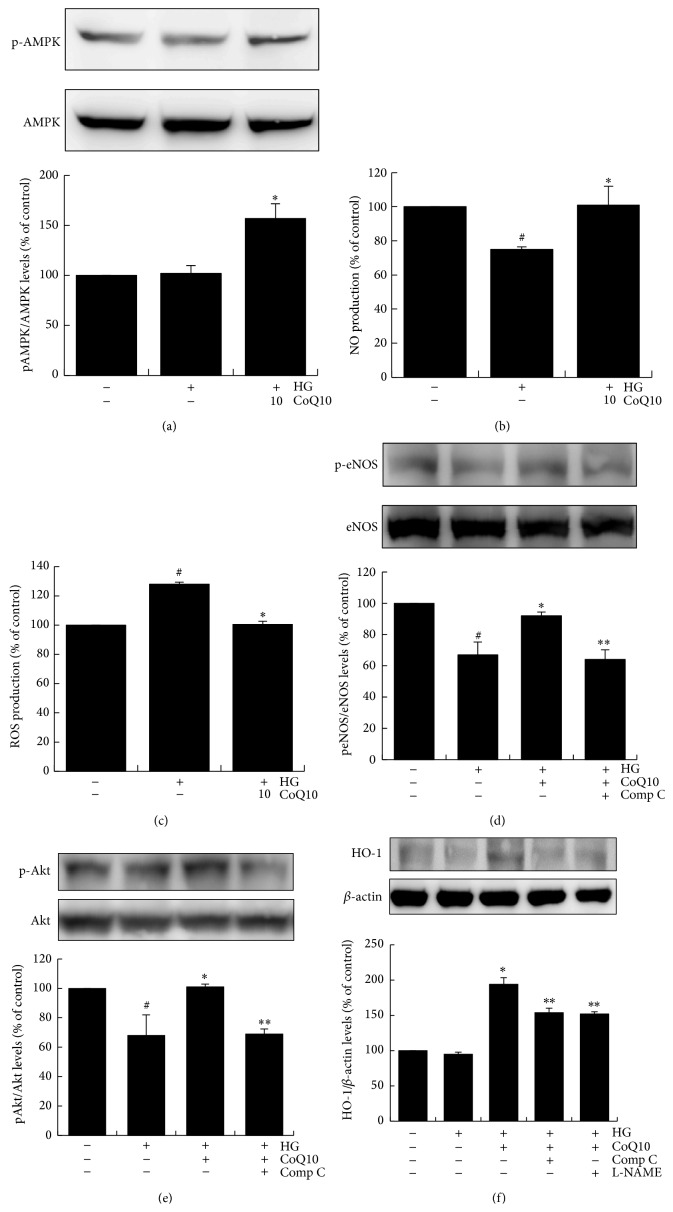
CoQ10 attenuates ROS and activates NO and AMPK pathway of EPCs under high glucose conditions. EPCs were incubated with CoQ10 (10 *μ*M) under high glucose conditions. (a) AMPK protein phosphorylation levels of EPCs were analyzed by western blot. Data are mean ± SE; *n* = 6. (b) NO production of EPCs was assessed by staining with NO fluorescent indicator 3-amino,4-aminomethyl-2′,7′-difluorofluorescein (DAF-FM) diacetate (10 *μ*M) for 30 min. (c) ROS production of EPCs was assessed by staining with DCFH-DA (10 *μ*M) for 20 min. The fluorescence intensity was measured using a fluorescent microplate reader. ((d), (e), and (f)) Expressions of Akt protein phosphorylation, eNOS protein phosphorylation, and HO-1 protein were assessed by western blot analysis. Comp C: component C, AMPK inhibitor; L-NAME, NO inhibitor. Data are mean ± SE; *n* = 4; ^#^ *P* < 0.05 versus control (5 mM glucose); ^*∗*^ *P* < 0.05 versus high glucose (HG); ^*∗∗*^ *P* < 0.05 versus HG-CoQ10.

To identify the possible mechanistic pathway of CoQ10 to recover EPC functions suppressed by high glucose, phosphorylated-AMPK expression was investigated in cultured EPCs exposed to high glucose. As shown in Figure 4(a), phosphorylated-AMPK expression was not enhanced in response to high glucose stimulation, but treatment with CoQ10 (10 *μ*M) significantly upregulated phosphorylated-AMPK expression.

We further examined whether CoQ10 reduces high glucose-stimulated NO and ROS production. The data shows that high glucose reduced NO production of EPCs by 28% and treatment with CoQ10 reversed NO production by 30% (Figure 4(b)). On the other hand, ROS production was promoted by high glucose medium (increased 28%), and high glucose promoted-ROS production was significantly attenuated by treatment with CoQ10 (Figure 4(c)).

Moreover, administration of CoQ10 upregulated phosphorylation of Akt, eNOS and increased HO-1 expressions of EPCs in high glucose conditions (Figures 4(d)–4(f)). Of note, these effects were significantly nullified by administration of AMPK inhibitor. Furthermore, the administration of a NO inhibitor (L-NAME) reduced the activation of CoQ10-promoted HO-1 in the high glucose environment. These results suggested CoQ10 could activate eNOS, AKT, and HO-1 expressions through the AMPK pathway in high glucose-induced EPCs.

### 3.6. CoQ10 Improves High Glucose-Suppressed EPC Function through AMPK, NO, and HO-1 Pathways

**Figure 5:**
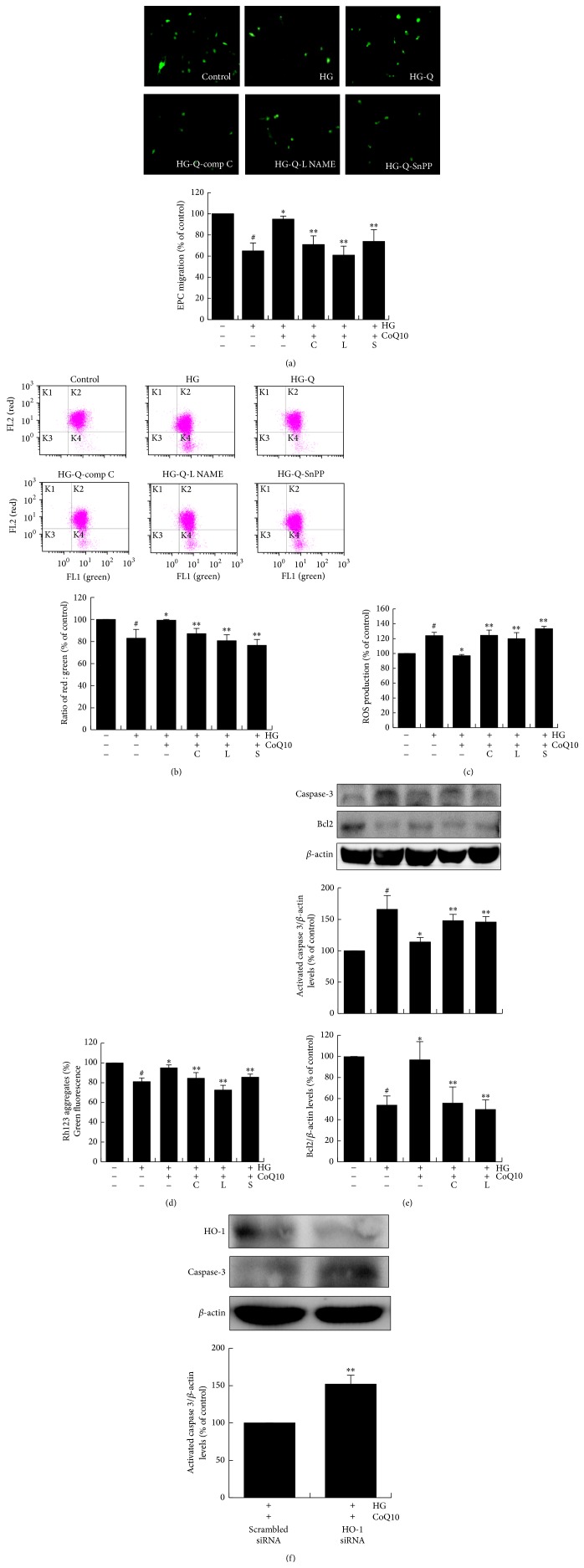
CoQ10 improves high glucose-induced EPCs dysfunction by upregulation of eNOS and HO-1. Cells were cultured with comp C (20 *μ*M), L-NAMEA (100 *μ*M), and SnPP IX (10 *μ*M) for 60 min before CoQ10 incubation under high glucose conditions. (a) EPC migration was measured by Boyden chamber assay. The migrated cells were stained with fluorescein isothiocyanate UEA-1 (lectin) (green) and counted under the fluorescence microscope. (b) Mitochondrial apoptosis was detected by JC-1. Loss of mitochondrial membrane potential (ΔΨ_m_) was assessed by the change in JC-1-derived fluorescence from red to green. The ratio of red/green fluorescence represented ΔΨ_m_ in EPCs. (c) ROS production of EPCs was assessed by staining with DCFH-DA. (d) The mitochondrial function was measured using Rh123 dye (5 mM), and the fluorescence intensity was measured at 485-nm excitation and 530-nm emission using a fluorescent microplate reader. (e) Expressions of activated-caspase 3 and Bcl2 protein were assessed by western blot. (f) Cells were transfected with scramble and HO-1 siRNA (10 nM), respectively, in CoQ10-treated EPCs under high glucose conditions, and the expressions of activated-caspase 3 and HO-1 protein were detected by western blot. Comp C: component C, AMPK inhibitor; L-NAME, NO inhibitor; Snpp IX, HO-1 inhibitor. Data are mean ± SE; *n* = 4; ^#^ *P* < 0.05 versus control (5 mM glucose); ^*∗*^ *P* < 0.05 versus high glucose (HG); ^*∗∗*^ *P* < 0.05 versus HG-CoQ10.

To confirm that HO-1, NO, and AMPK pathways are involved in the effects of CoQ10 on cell functions suppressed by high glucose, EPCs were pretreated with compound C (AMPK inhibitor, 10 *μ*M), L-NAME (NO inhibitor, 100 *μ*M), and SnPP IX (HO-1 inhibitor, 5 *μ*M) for 1 hour before CoQ10 treatment. As shown in Figure 5(a), treatment of EPCs with compound C, L-NAME, and SnPP IX reduced CoQ10-improved migratory capacity by 25%, 33%, and 23%, respectively. In addition, administration of compound C, L-NAME, and SnPP IX significantly reversed CoQ10-improved mitochondrial apoptosis, mitochondrial membrane potential, and ROS production (Figures 5(b)–5(d)).

Western blot analysis revealed that high glucose increased activated-caspase 3 expression, and treatment with CoQ10 suppressed this effect. Additionally, CoQ10-induced activated-caspase 3 protein was significantly downregulated by AMPK and NO inhibitors (34% and 32%, resp.) after CoQ10 treatment in high glucose conditions (Figure 5(e)). Moreover, the antiapoptotic protein Bcl-2 was downregulated by high glucose stimulation but recovered by CoQ10 treatment. However, this upregulation was reduced by AMPK and NO inhibitors by 40% and 45%, respectively. In addition, administration of HO-1 siRNA (10 nM) enhanced CoQ10 suppressed activated-caspase 3 protein in high glucose medium (Figure 5(f)). These data indicate that CoQ10 improved EPC function and attenuated cellular apoptosis through AMPK, NO, and HO-1 pathways.

### 3.7. CoQ10-Treated EPCs Transplantation Improves Hindlimb Perfusion

**Figure 6:**
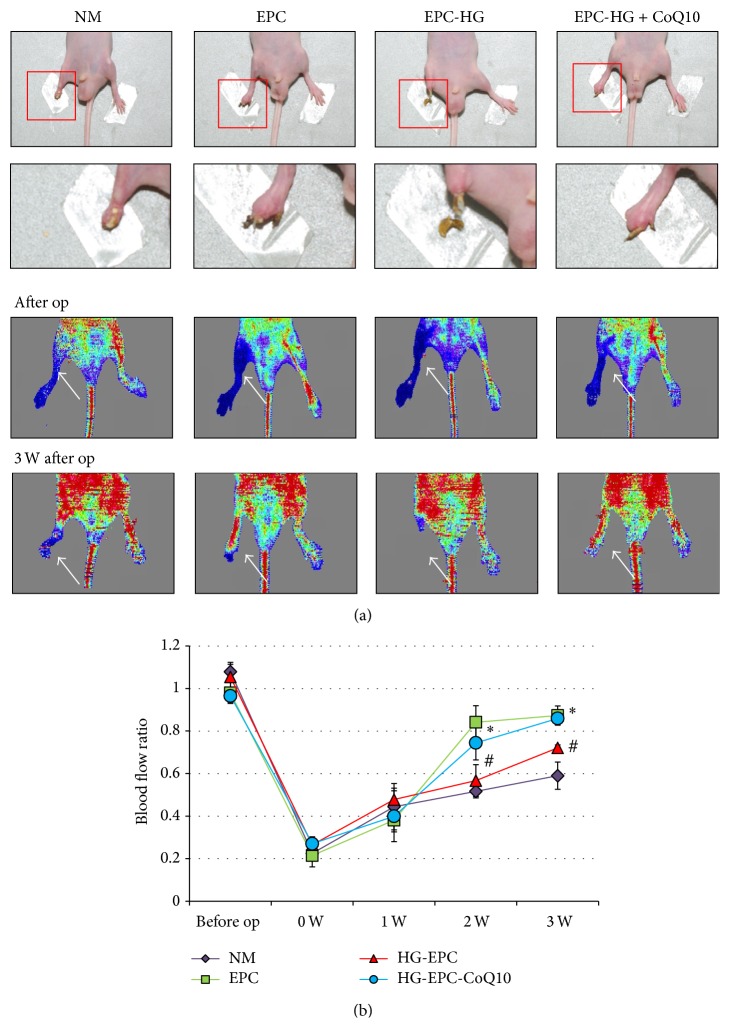
Effect of CoQ10-treated EPCs in high glucose medium transplantation on hindlimb perfusion. (a) Serial laser Doppler analyses of hindlimb perfusion revealed before and 3 weeks after hindlimb ischemia surgery in nude mice, which received a transplant with normal saline, EPCs, high glucose-treated EPCs, and high glucose-treated EPC incubated with CoQ10. Low or no perfusion is displayed as blue, whereas the highest perfusion is displayed as red. Arrows indicate ischemic (right) limb after hindlimb ischemia surgery. (b) Quantification analysis of perfusion recovery by laser Doppler perfusion imaging rations (ischemic/normal hindlimb) over time in the different groups. Results are mean ± SE; *n* = 4; ^#^ *P* < 0.05 versus control (5 mM glucose); ^*∗*^ *P* < 0.05 versus high glucose (HG).

To further elucidate whether CoQ10 improves EPCs' angiogenic function, EPCs, high glucose-treated EPCs, and high glucose-treated EPCs incubated with CoQ10 were separately transplanted into ischemic hindlimbs in nude mice. EPCs were labeled with PKH26 fluorescence after being cultured with high glucose or high glucose treated with CoQ1K0 (10 *μ*M). Blood flow of normal saline mice kept constant throughout the study, by about 50% of that measured in the nonischemic limb (3 weeks after operation). By contrast, mice transplanted with EPC were cultured with high glucose and CoQ10 medium and had a better blood flow recovery than only high glucose cultured EPC (Figure 6). These data suggest that blood flow in ischemic hindlimbs could be improved by transplantation with healthy EPCs and CoQ10-treated EPCs cultured under high glucose conditions.

## 4. Discussion

The defects in EPC functions and behavior may underlie some of the vascular complications associated with diabetes, such as endothelial dysfunction, that predispose a diabetic patient to diffuse atherosclerosis and impaired neovascularization after ischemic events [6, 16, 17]. Consequently, the idea of using EPCs as a therapeutic agent has grown in popularity. To the best of our knowledge, this is the first study to show the benefit of CoQ10 on high glucose-suppressed EPC functions. Administration of CoQ10 improved EPC functions, decreased ROS, increased NO production, and attenuated cellular apoptosis, with the mechanism of these effects being shown to involve AMPK, eNOS, and HO-1 pathways. Transplantation of CoQ10-treated EPCs under high glucose conditions into ischemic hindlimbs improved blood flow recovery more than in those that received only high glucose-incubated EPCs. Our findings provide novel evidence that CoQ10 could be a potential therapeutic agent to diminish high glucose-attenuated EPC angiogenic functions in diabetic patients.

Diabetes mellitus has reached epidemic proportions worldwide and is associated with a large economic burden and markedly increased risk of cardiovascular diseases. A large body of evidence suggests a causal link between diabetic hyperglycemia and the development of vascular complications [18]. However, the mechanisms that underlie diabetic hyperglycemia-induced vascular complications remain to be determined. Circulating EPCs are derived mainly from the monocyte/macrophage lineage, and are capable of forming new blood vessels through a process of vasculogenesis [3, 4]. Clinical studies have reported that EPCs are markedly decreased in diabetic patients, and EPCs from diabetic patients show reduced capacity to induce angiogenesis in vitro [19]. Importantly, impaired postischemic EPC mobilization in diabetic animals has been demonstrated previously [20]. The impairment of EPC functions and behavior may promote some of the vascular complications associated with diabetes that predispose diabetic patients to diffuse atherosclerosis and attenuated neovascularization after ischemic events.

It is well known that a causal link between diabetic hyperglycemia and the development of macrovascular and microvascular complications. Macrovascular complications include coronary artery disease, atherosclerosis and peripheral vascular disease, and microvascular complications include retinopathy, nephropathy and neuropathy [21]. Recent evidence suggests that NO has a crucial role in maintaining EPC and endothelial cells function [22]. Long-term exposure to high glucose conditions might enhance EPCs senescence and decrease cell numbers and functional competencies of EPCs via NO-related mechanisms [6]. Moreover, high glucose induces cytochrome-C release and promotes apoptosis of endothelial cells due to downregulation of NO bioavailability [23]. In line with previous reports, our results indicated that high glucose impaired Akt/eNOS activity of EPCs, as well as NO production, and administration of CoQ10 recovered eNOS activation through the AMPK pathway.

CoQ10, also known as ubiquinone-10 or ubiquinol-10, is well understood as an important component in the oxidative phosphorylation of mitochondria and the production of adenosine triphosphate [24]. CoQ10 is stored in the mitochondria of cells as a mobile lipophilic electron carrier and regulates respiratory chain activity. In addition, CoQ10 involves NAD(P)H-oxidoreductase-dependent reactions such as in NO synthesis in Golgi and plasma membranes [25]. Previous studies have indicated that CoQ10 attenuates cellular apoptosis in corneal fibroblasts by inhibition of mitochondrial depolarization [13, 26] and prevents HUVEC apoptosis through suppression of mitochondria dependent caspase 3 protein. However, there has been no report investigating the direct effect of CoQ10 on EPC in vitro. In our study, we described that administration of CoQ10 improved high glucose-suppressed EPC function and reversed apoptosis of EPCs by enhancement of mitochondrial function, enhanced Bcl-2 expression, and downregulation of caspase 3, which suggest that CoQ10 might improve EPC functional impairment and survival in diabetic patients. Additionally, CoQ10 was shown to reduce ROS production under high glucose conditions and improve EPC function by upregulation of eNOS and HO-1 through the AMPK pathway. These findings are consistent with previous reports and suggest that activation of CoQ10 might reduce hyperglycemia-induced mitochondrial dysfunction and promote mitochondrial biogenesis in EPCs exposed to high glucose [27, 28].

CoQ10, an antioxidant, scavenges free radicals and inhibits apoptosis in the mitochondria of cells. A recent study indicated that CoQ10 inhibited caspase 3 dependent apoptosis which was regulated by mitochondrial signaling in high glucose-treated HUVECs [29]. Moreover, CoQ10 displayed an antiapoptotic effect by suppressing mitochondrial membrane depolarization, cytochrome-C release, and caspase activation [14, 30]. CoQ10 has the ability to mediate oxidative stress and prevent diabetic endotheliopathy by eNOS activation [30]. In the current report, we showed that high glucose impaired Akt/eNOS phosphorylation and NO production, reduced EPC functional activity, and increased cell apoptosis. Treatment with CoQ10 improved Akt/eNOS phosphorylation and NO production and reversed high glucose-induced EPC damage.

Besides, Li et al. indicated that AMPK transgenic mice were resistant to hyperglycemia-induced impairment in endothelium dependent relaxation and reendothelialization of injured carotid arteries through HO-1 [31]. In our study, we found that HO-1 expression was upregulated by CoQ10, and administration of SnPP IX (HO-1 inhibitor) blocked CoQ10-improved EPCs' migration and apoptosis. These results extended previous studies suggesting that HO-1 overexpression in EPCs promoted reendothelialization and inhibited neointimal hyperplasia in injured vessels [32]. The beneficial effects may provide a new viewpoint for the use of CoQ10 therapy for its vascular protective properties in diabetic patients with critical limb ischemia or provide a clinical incentive to improve dysfunctional EPCs before cell therapy.

## 5. Conclusion

**Figure 7:**
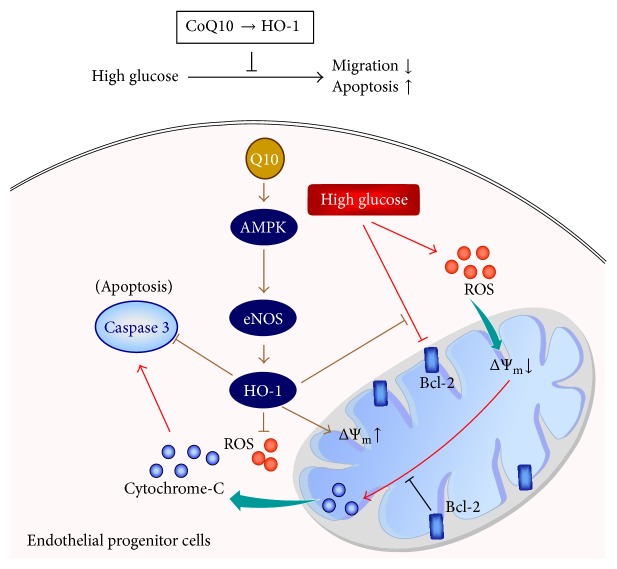
The schematic diagram summarizes possible mechanisms by which CoQ10 reduces hyperglycemia-induced endothelial progenitor cell damage. In high glucose condition, CoQ10 improved EPCs migration and apoptosis by ROS and caspase 3 downregulation and mitochondrial membrane potential (ΔΨ_m_) and Bcl-2 upregulation through HO-1 pathway.

This study provides a notion that CoQ10 has beneficial effects in high glucose-induced EPC apoptosis and dysfunction in vitro. These data may clarify the underlying mechanisms responsible for the benefit of CoQ10 on the treatment of diabetic vasculopathy and cardiovascular diseases (Figure 7).
